# A Potential Peptide From Soy Cheese Produced Using *Lactobacillus delbrueckii* WS4 for Effective Inhibition of SARS-CoV-2 Main Protease and S1 Glycoprotein

**DOI:** 10.3389/fmolb.2020.601753

**Published:** 2020-12-11

**Authors:** Rounak Chourasia, Srichandan Padhi, Loreni Chiring Phukon, Md Minhajul Abedin, Sudhir P. Singh, Amit Kumar Rai

**Affiliations:** ^1^Institute of Bioresources and Sustainable Development (DBT-IBSD), Regional Centre, Sikkim, India; ^2^Center of Innovative and Applied Bioprocessing (DBT-CIAB), Mohali, India

**Keywords:** *Lactobacillus delbrueckii* WS4, soy cheese, peptides, main protease, S1 glycoprotein

## Abstract

The COVID-19 pandemic caused by novel SARS-CoV-2 has resulted in an unprecedented loss of lives and economy around the world. In this study, search for potential inhibitors against two of the best characterized SARS-CoV-2 drug targets: S1 glycoprotein receptor-binding domain (RBD) and main protease (3CL^Pro^), was carried out using the soy cheese peptides. A total of 1,420 peptides identified from the cheese peptidome produced using *Lactobacillus delbrueckii* WS4 were screened for antiviral activity by employing the web tools, AVPpred, and meta-iAVP. Molecular docking studies of the selected peptides revealed one potential peptide “KFVPKQPNMIL” that demonstrated strong affinity toward significant amino acid residues responsible for the host cell entry (RBD) and multiplication (3CL^pro^) of SARS-CoV-2. The peptide was also assessed for its ability to interact with the critical residues of S1 RBD and 3CL^pro^ of other β-coronaviruses. High binding affinity was observed toward critical amino acids of both the targeted proteins in SARS-CoV, MERS-CoV, and HCoV-HKU1. The binding energy of KFVPKQPNMIL against RBD and 3CL^pro^ of the four viruses ranged from −8.45 to −26.8 kcal/mol and −15.22 to −22.85 kcal/mol, respectively. The findings conclude that cheese, produced by using *Lb. delbrueckii* WS4, could be explored as a prophylactic food for SARS-CoV-2 and related viruses. In addition, the multi-target inhibitor peptide, which effectively inhibited both the viral proteins, could further be used as a terminus a quo for the *in vitro* and *in vivo* function against SARS-CoV-2.

## Introduction

In December 2019, a contagious respiratory illness emerged in the Wuhan city of China. This outbreak is rapidly evolving and brings concerns about the potential severe harms across the globe. With increasing cases of infections and reported death toll every day, millions of people are suffering in the insufficiency of specific strategies to treat or prevent such attacks. The causative organism of this outbreak is a novel viral entity belonging to the genera of β-coronavirus, which was eventually identified by the International Committee on Taxonomy of Viruses (ICTV) as Severe Acute Respiratory Syndrome Coronavirus 2 or SARS-CoV-2. Researchers are focusing on the development of vaccines and bioactive metabolites from natural sources as therapeutics (Corbett et al., 2020; Izda et al., 2020; Padhi et al., 2020). Among vaccines, two leading mRNA-based SARS CoV-2 vaccine candidates, mRNA-1273, and BNT162b1-b2 have demonstrated a robust anti-SARS-CoV-2 neutralizing antibody response in clinical trials (Anderson et al., 2020; Mulligan et al., 2020). The recent developments regarding this disease have identified vital viral proteins, the spike glycoprotein (S1), and the main protease (3CL^pro^) that are central to the virus entry and its life cycle inside the host (Hall and Ji, 2020). These human infecting β-coronavirus proteins contain conserved motifs and are considered as the best-characterized drug targets (Nadeem et al., 2020). The main protease consists of two identical subunits that together form two active sites and play an essential role in the processing of the RNA translated polyproteins (Ullrich and Nitsche, 2020; Zhang et al., 2020). On the other hand, S1 glycoprotein in a receptor-mediated interaction binds with human ACE2 (angiotensin-converting enzyme 2), and this facilitates the virus entry into the cell. Moreover, the receptor-binding domain (RBD) associated with S1 glycoprotein is a crucial factor in mediating the interaction with the human ACE2 (Prajapat et al., 2020; Wu et al., 2020). There is an urgent need to search for potent inhibitors that could effectively block these target proteins and lead to the discovery and development of novel therapeutics.

Fermented soy milk products formed as a result of controlled fermentation by lactic acid bacteria (LAB) have gained increasing attention for its nutritional quality and health-promoting attributes (Sirilun et al., 2017). In addition to nutritional essentials of the soy, fermentation offers a means of delivering specific indigenous microbes into the intestine where they can accomplish several functionalities like modulating the immune response, improving epithelial barrier function, or modifying indigenous gut microbiota (Nagino et al., 2018). The most common LAB responsible for soy milk fermentation belongs to the genus *Lactobacillus* that is ubiquitous in several niches. It has a safe history in the food processing industry. A majority of LAB have received the generally recognized as safe (GRAS) status by U.S. Food and Drug Administration (FDA) (Montel et al., 2014). Nevertheless, the current research is focused on bioactive peptides released during fermentation of soy milk, with products including ripened cheeses and yogurts becoming subjects of numerous studies. Emerging pieces of evidence support the potential of these peptides in preventing many infectious diseases and adverse health conditions (Pihlanto, 2006; Cicero et al., 2017). Furthermore, *in silico* approaches such as virtual screening and molecular docking have become very popular in designing and exploring these bioactive peptides as potential ligands to the protein targets linked with numerous diseases (Chi and Vargas, 2020).

In the present study, peptides identified from soy cheese fermented using *Lactobacillus delbrueckii* WS4, isolated from a traditional *chhurpi* cheese, were screened for *in silico* antiviral activity. Twenty-three selected peptide sequences were examined for binding affinity toward critical residues of SARS-CoV-2 RBD protein and important catalytic residues of the SARS-CoV-2 3CL^pro^ enzyme using molecular docking. The conformations of the peptide-receptor showed promising binding affinity toward the targeted residues on the surface of both the viral proteins. Selected peptide demonstrating the highest affinity for both SARS-CoV-2 proteins were docked with RBD and 3CL^pro^ of other β-coronaviruses including SARS-CoV, MERS-CoV, and HCoV-HKU1 to examine the potential broad-spectrum binding affinity of the peptide. This study represents a novel strategy toward the search for peptide-based therapeutics against SARS-CoV-2 and related viruses by investigating the inhibitory action of fermented soy milk-derived peptides on protein molecules responsible for host cell entry and viral replication.

## Materials and Methods

### Sample Details

Proteolytic *Lactobacillus delbrueckii* WS4, isolated from curd obtained during traditional *chhurpi* production was revived by passing twice in de Man Rogosa and Sharpe (MRS) broth (Sigma-Aldrich, USA). Yellow soybean seeds for soy cheese production were purchased from the local market (Gangtok, Sikkim). Fermentation of freshly prepared soy milk was followed by the production of fresh soy cheese. The identification of peptides in soy cheese water-soluble extract (WSE) was done by LC-MS/MS analysis. PicoFrit column (60 cm, 360 μm outer diameter, 75 μm inner diameter, 10 μm tip) filled with 3.0 μm of C18-resin (Dr. Maeisch, Germany) was used to resolve peptide mixture. EASY-nLC 1000 system coupled to Thermo Fisher-Q Exactive (Thermo Fisher Scientific, USA) equipped with nano-electrospray ion source was used for mass spectrometric analysis. A total of 1420 peptides originating from different soy proteins, including glycinin, β-conglycinin, proglycinin, lectins, and trypsin inhibitors, were identified (unpublished data).

### Target Receptors for SARS-CoV-2 Inhibition

Two major SARS-CoV-2 proteins, the receptor-binding domain (RBD) of S1 glycoprotein and the main protease (3CL^pro^) are involved in the host cell infection and viral replication, respectively. These proteins were selected as peptide targets in this investigation, and X-ray crystallographic structure of the proteins were retrieved from RCSB PDB (https://www.rcsb.org/). A 193 amino acid long (THR333.GLY526) 3D structure of RBD was processed from the crystal structure of RBD-ACE2 complex (resolution: 2.45 Å; PDB ID: 6M0J). The RBD comprises a core region that is stabilized by three pairs of cysteine residues, five beta pleated sheets organized in anti-parallel manner and a functional motif region known as a receptor-binding motif (RBM). RBM is supported by two alpha helices, two beta sheets, and connecting loops (Tai et al., 2020).

The second viral protein is the non-structural protein, a 3-chymotrypsin-like cysteine protease (3CL^pro^). This enzyme plays an essential role in the processing of polyproteins translated from viral RNA. Inhibition of the activity of this enzyme could block viral replication. Since the cleavage specificity of 3CL^pro^ differs from that of human proteases, inhibitors of this enzyme are unlikely to be toxic to the human body (Zhang et al., 2020). The 3D structure of unliganded 3CL^pro^ with 1.25 Å resolutions was retrieved (PDB ID: 6LU7). The enzyme is organized into three domains; domain I (8–101), II (102–184), and III (201–303). Domain I and II have antiparallel β barrel structure, while Domain III is a large antiparallel globular structure containing five α-helices. Doman III is connected to domain II by a loop (185–200).

### Selection of Peptides by *in silico* Antiviral Activity Prediction

LC-MS/MS identified soy cheese peptides were screened for antiviral activity using the web servers AVPpred (http://crdd.osdd.net/servers/avppred/submit.php) (Thakur et al., 2012) and meta-iAVP (http://codes.bio/meta-iavp/) (Schaduangrat et al., 2019). AVPpred relies on a dataset of peptides (1,245 no.s) that have been experimentally checked for antiviral activity targeting important human viruses like influenza, HIV, HCV and SARS, etc. The prediction of antiviral activity by AVPpred was based on Support Vector Machine (SVM) models followed by 5-fold cross-validation. Sequence features, amino acid composition and effect of hydrophobic and amphiphilic amino acid residues in bioavailability and activity were analyzed. The other criteria include alignment of the query sequence with peptides in antiviral and non-AVP databases using BLASTP, analysis of physicochemical properties like overall charge, size, and secondary structure of the query peptide. The query peptides were analyzed by MEME (Multiple Em for Motif Elicitation)/MAST (Motif Alignment and Search Tool) (Thakur et al., 2012) for identification of AVP conserved motifs. Meta-iAVP uses “effective feature presentation,” extracted from a set of prediction scores derived from various machine learning algorithms and types of features (Schaduangrat et al., 2019). The peptide sequences predicted to be antiviral by both the web tools were *in silico* tested for toxicity using the ToxinPred web-server (Gupta et al., 2013), followed by molecular docking.

### Molecular Docking Against Target SARS-CoV-2 Proteins

Construction of 3D structure of the selected peptides was done using PEPFOLD (Lamiable et al., 2016). PDB structure files of the target receptors and selected peptides were imported to Discovery Studio (DS). This was followed by cleaning and processing of the structures using the “prepare protein” tool. This tool is used for addition of missing residues and polar hydrogens, removal of unwanted water molecules and hetero atoms, inserting missing loops, charge assignment, and fixing CHARMM force field. The rigid-body protein-protein docking program DS-ZDOCK was used to study the possible interactions between peptides and target receptors. A Fast Fourier Transform (FFT) search algorithm, DS-ZDOCK, identifies docked conformations using a pair-wise shape complementarity function and scores hits based on atomic contact energies. Peptide-receptor conformations with the highest DS-ZDOCK scores were subjected to CHARMM and desolvation energy-based re-ranking of DS-ZDOCK predictions using the DS-RDOCK algorithm.

### Comparative Study of Peptide Interactions With RBD and 3CL^pro^ of β-Coronaviruses

Soy cheese peptide showing highest binding affinity toward RBD and 3CL^pro^ of SARS-CoV-2 was selected for molecular docking against similar proteins of other β-coronaviruses. X-ray crystallographic RBD structures of SARS-CoV (PDB ID: 2AJF), MERS-CoV (PDB ID: 6C6Z), and HCoV-HKU1 (PDB ID: 5GNB) were pre-processed, and interaction of each RBD with the selected peptide was studied using ZDOCK and RDOCK programs. Similarly, molecular docking of the selected peptide was performed individually with 3CL^pro^ of SARS-CoV (PDB ID: 1UK4), MERS-CoV (PDB ID: 5WKK), and HCoV-HKU1 (PDB ID: 3D23). Molecular docking experiments and energy calculations were performed using BIOVIA Discovery Studio Client v20.1.0.19.295, 2020 (BIOVIA Solutions, San Diego, California, US).

## Results

### *In silico* Antiviral Prediction of Soy Cheese Peptides

**Figure 1:**
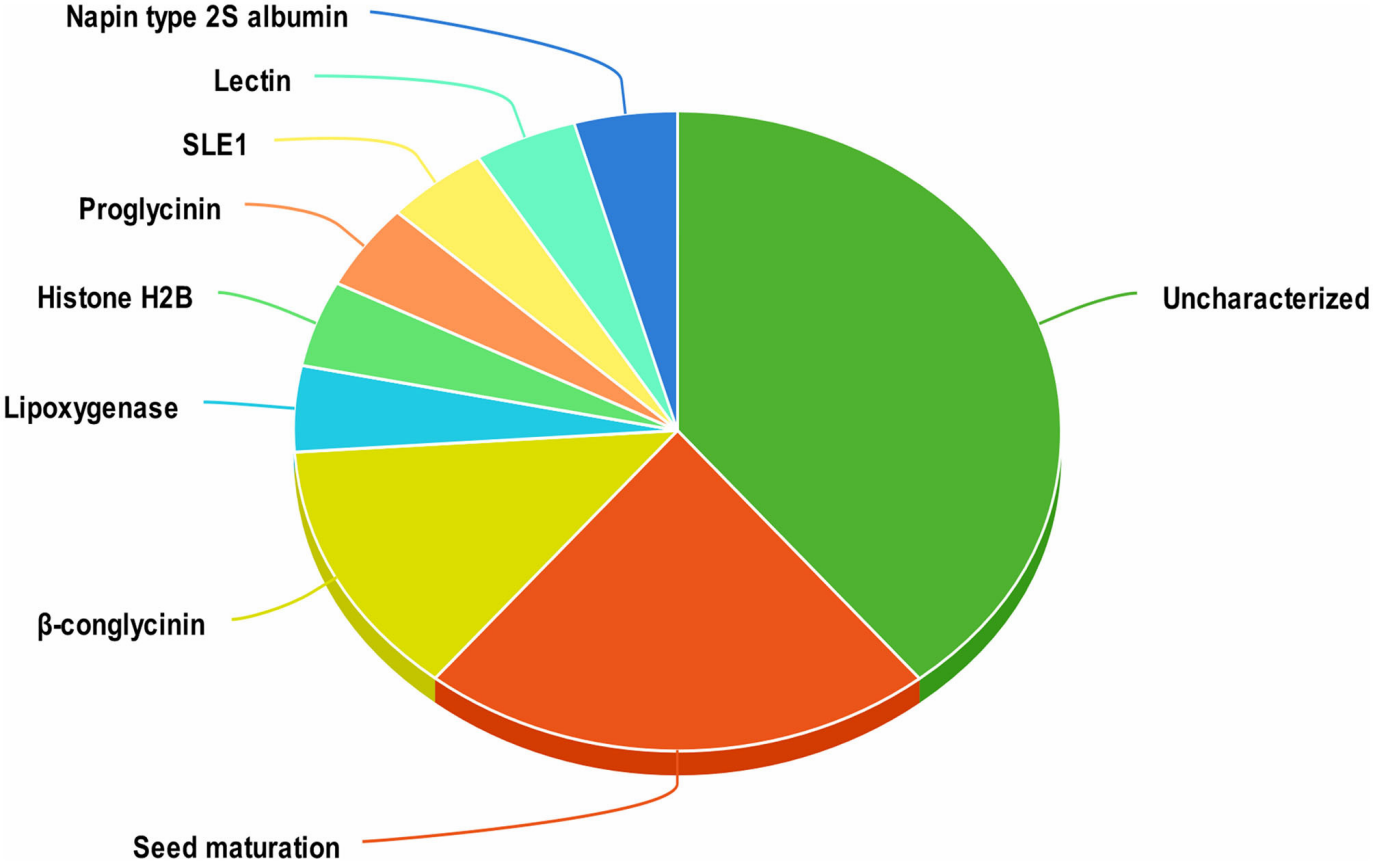
Distribution of antiviral peptides among their proteins of origin in soy cheese produced using *Lactobacillus delbrueckii* WS4.

A total of 1420 peptides were identified by LC-MS/MS analysis of aqueous extracts of soy cheese prepared using *Lb. delbrueckii* WS4. Screening of these peptides by two different predictive web servers resulted in the selection of 23 peptides having antiviral activity. Most of the selected peptides had an uncharacterized soy protein source, while other peptides were derived from a variety of soy proteins, including β-conglycinin, proglycinin, seed maturation protein, and lectin (Figure 1).

The physiochemical parameters of the selected peptides are detailed in Table 1. The computed molecular weight of the peptides ranged from 0.9 to 4.03 kDa. The isoelectric point of a protein is the measure of its charge with reference to the pH of its environment. The pI of SARS-CoV-2 RBD and 3CL^pro^ was calculated to be 8.05 and 5.95, respectively. Theoretical pI of the selected peptides ranged from 3.62 to 11.17, indicating the presence of both negatively and positively charged peptides. GRAVY values of all except three peptides were negative, indicating a hydrophilic nature of the peptides. Prediction of toxicity using ToxinPred tool revealed that all selected peptides were non-toxic.

**Table 1:** Computed physicochemical features of predicted antiviral peptide sequences.

**Peptide sequence(s)**	Mol. Wt.^*^ (kDa)	Th. pI^*^	GRAVY^*^	**Toxicity Y/N**
ALEIGTKSL	0.93	6.05	0.51	N
ATAQTVGQKAVDQSDASAIQAAEVRAT	2.68	4.56	−0.20	N
ATAQTVGQKAVDQSDASAIQAAEVRATGSN	2.94	4.56	−0.34	N
AVRLVLPGELAKHAVSE	1.78	6.80	0.47	N
DAITIGEALEASAIAGASDKPVDESD	2.54	3.62	−0.06	N
DRPSIGNLAGANSLLNALPEEVIQHTFNLKSQQ	3.57	5.45	−0.40	N
ENKILQISGE	1.13	4.53	−0.63	N
ENLGGIGEKRE	1.20	4.79	−1.39	N
FLEHAFSVDKQIAKNLQGENEGEDKGAIVTVKGGL	3.74	5.01	−0.38	N
GENEGEDKGAIVTVKGGLSVIKPPTDEQQQRPQ	3.50	4.66	−1.10	N
GIEIDESKFKIT	1.37	4.68	−0.32	N
GLSVIKPPTDEQQQRPQ	1.92	6.07	−1.38	N
GVIGSMFKA	0.90	8.75	1.07	N
IENLIKSQSESYFVDAQPQQKEEGN	2.88	4.25	−1.21	N
KFVPKQPNMIL	1.31	10.00	−0.07	N
KIMDNQSEQLE	1.33	4.14	−1.40	N
KRGVIGSMFK	1.12	11.17	−0.05	N
LSVISPKWQE	1.18	6.00	−0.25	N
MQGGKKAGESIKETAANIGASAKAGME	2.63	8.19	−0.51	N
NVISQIPSQVQELAFPGSAQAVEKLLKNQRESYFVD	4.03	4.87	−0.29	N
PSPPSVKARIL	1.16	11.01	−0.04	N
SEDEAVRVAYEHGSPLEGGKIADSQPVDLFSSAH	3.59	4.43	−0.52	N
SQVQELAFPGSAQAVEKLLKNQRESYFVD	3.28	4.87	−0.50	N

* *GRAVY, Grand average of hydropathy; Mol. Wt., Molecular weight; Th. pI, Theoretical isoelectric point*.

### Molecular Docking Against SARS-CoV-2 Protein Targets

**Figure 2:**
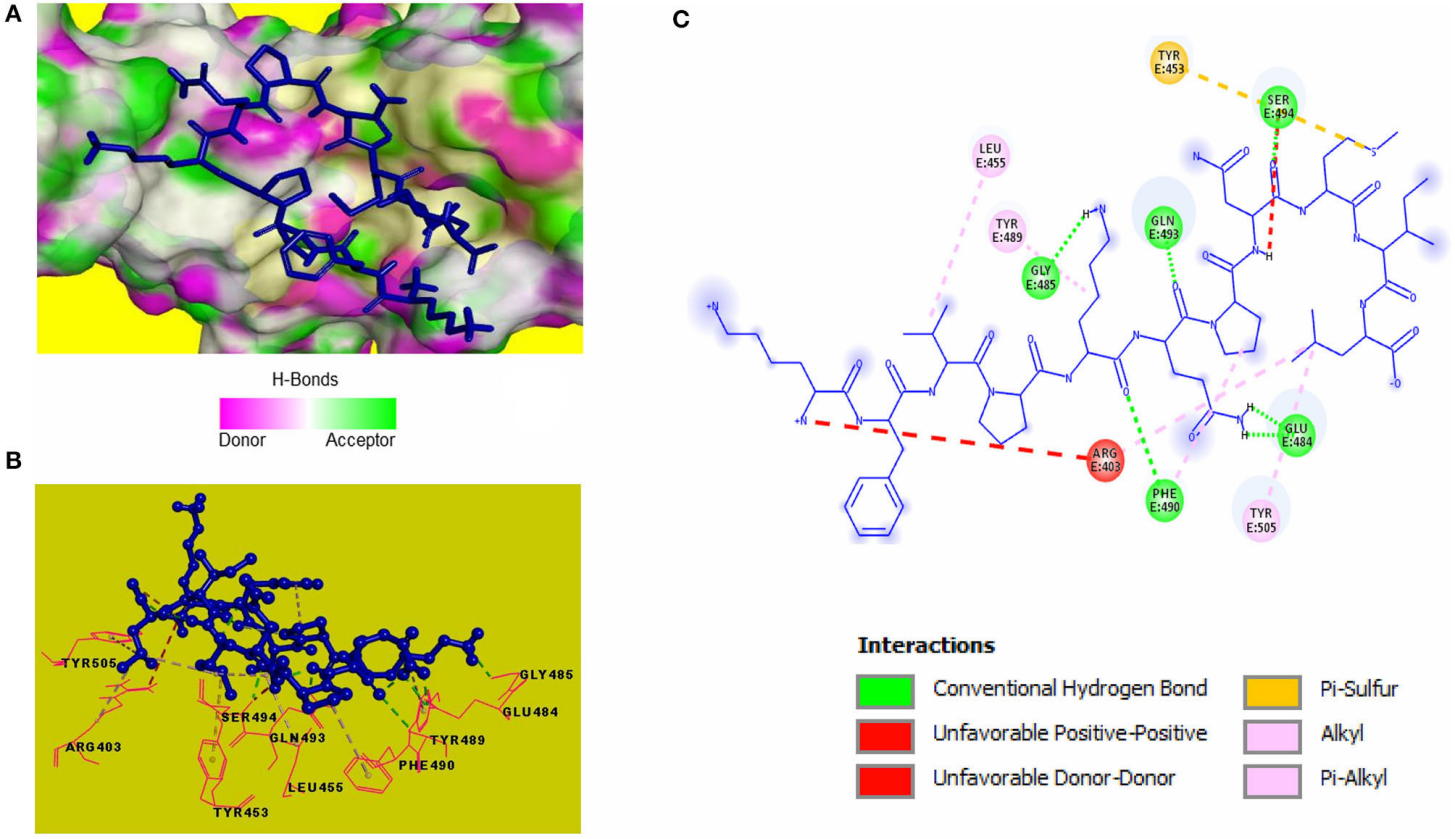
Receptor-ligand interactions between SARS-CoV-2 RBD and KFVPKQPNMIL. **(A)** Illustration showing molecular docking of KFVPKQPNMIL with RBD of S1 glycoprotein. RBD surface is represented by H-bond donor and acceptor atoms. **(B)** 3D interpretation representing structural interaction between peptidyl and RBM residues. **(C)** 2D diagram of peptide-RBD interactions including hydrogen bonds, Pi-Sulfur, Alkyl, and unfavorable interactions.

Blind molecular docking of the selected 23 peptides with SARS-CoV-2 RBD revealed that 14 peptides interacted with the RBM motif. Among these, 4 peptides were able to interact with critical amino acids within the RBM. The peptide, KFVPKQPNMIL, interacted with residues of the RBM with strong covalent, hydrophobic, and electrostatic bonds (Table 2). Conventional hydrogen bonds and alkyl interactions were observed with three critical amino acids, including a hydrogen bond with GLN493 (Figure 2). The predicted ZDOCK score and binding energy (E_RDOCK) of the KFVPKQPNMIL-RBD docked complex were 10.2 and −8.45 kcal/mol, respectively.

**Table 2:** Details of non-bond interactions between KFVPKQPNMIL and target protein residues of SARS-CoV-2.

**Peptidyl residue**	**Target protein residue**	**Interaction type**	**Distance (Å)**
SARS-CoV-2 RBD^*^			
LYS5	PHE490	Conventional hydrogen	2.98
GLN6	GLN493	Conventional hydrogen	2.00
ASN8	SER494	Conventional hydrogen	2.35
LYS5	GLY485	Conventional hydrogen	2.67
GLN6	GLU484	Conventional hydrogen	2.32
MET9	TYR453	Pi-Sulfur	5.63
LEU11	ARG403	Alkyl	5.42
VAL3	LEU455	Alkyl	4.86
LYS5	TYR489	Pi-Alkyl	5.35
PRO7	PHE490	Pi-Alkyl	5.11
LEU11	TYR505	Pi-Alkyl	5.09
**SARS-CoV-2 3CL**^pro^^*^			
LEU11	GLY143	Conventional hydrogen	2.03
LEU11	SER144	Conventional hydrogen	2.68
LEU11	CYS145	Conventional hydrogen	2.24
LEU11	CYS145	Conventional hydrogen	3.05
MET9	GLU166	Conventional hydrogen	2.07
GLN6	ALA191	Conventional hydrogen	2.44
PRO7	GLN192	Conventional hydrogen	2.07
LYS1	THR25	Conventional hydrogen	1.85
LYS1	CYS44	Conventional hydrogen	2.67
ASN8	VAL186	Conventional hydrogen	2.98
ILE10	GLN189	Conventional hydrogen	2.17
ILE10	HIS41	Pi-Sigma	3.71
PHE2	MET49	Pi-Sulfur	4.71
ILE10	MET49	Alkyl	4.91
PRO7	PRO168	Alkyl	4.59
PRO7	ALA191	Alkyl	4.11
LEU11	HIS163	Pi-Alkyl	5.42

* *RBD, Receptor binding domain of S1 spike protein; 3CLpro, 3-chymotrypsin-like cysteine protease*.

**Figure 3:**
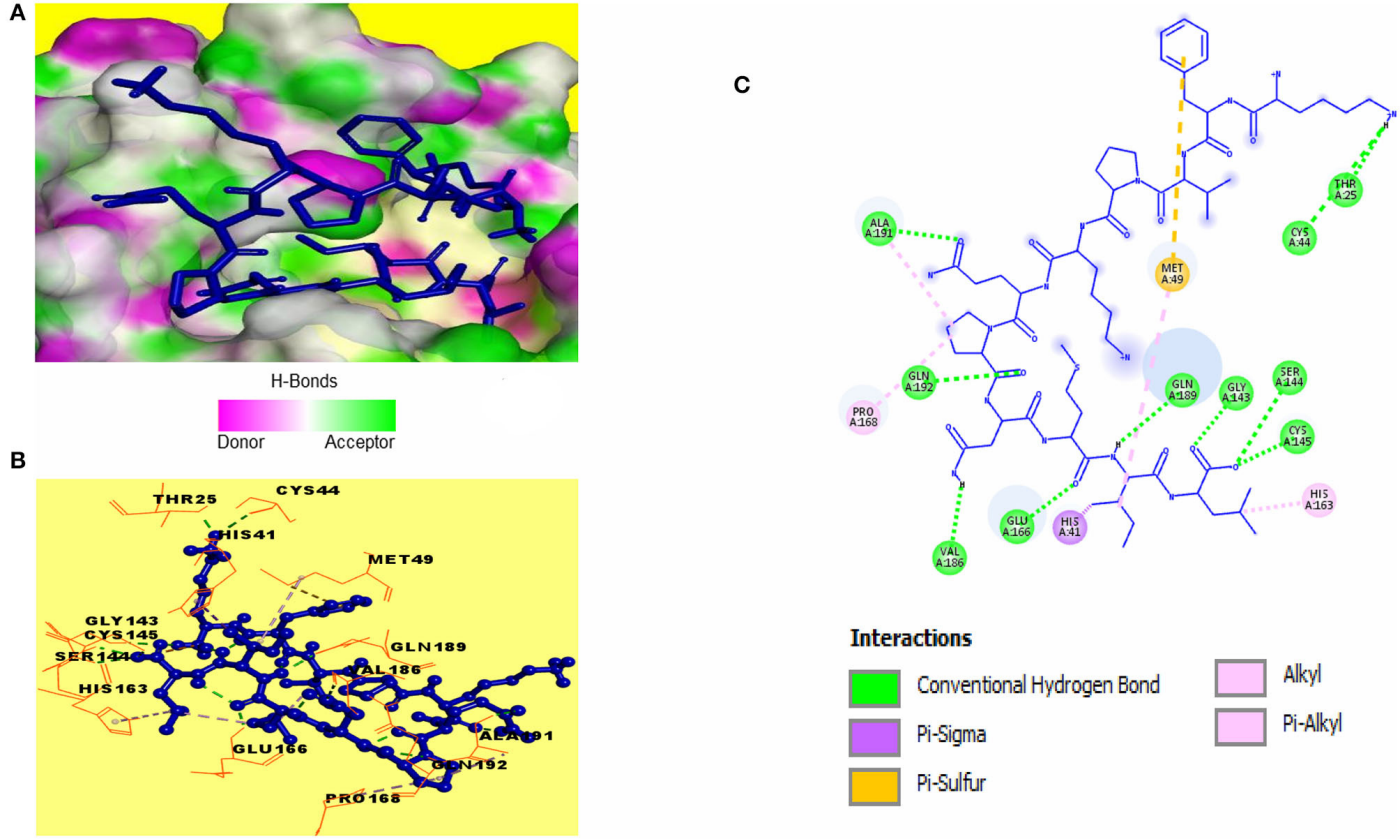
Receptor-ligand interactions between SARS-CoV-2 3CL^pro^ and KFVPKQPNMIL. **(A)** Illustration showing molecular docking of KFVPKQPNMIL with active site of 3CL^pro^. 3CL^pro^ surface is represented by H-bond donor and acceptor atoms. **(B)** 3D interpretation representing structural interaction between peptidyl and 3CL^pro^ residues. **(C)** 2D diagram of peptide-3CL^pro^ interactions including hydrogen bonds, Pi-Sulfur, Pi-Sigma, and Alkyl bonds.

Catalytic residues of SARS-CoV-2 3CL^pro^ for substrate binding include THR45, MET49, PHE140, ASN142, MET165, GLU166, HIS172, ASP187, ARG188, GLN189, and the CYS145-HIS41 dyad (Macchiagodena et al., 2020). Molecular docking of the selected peptides with SARS-CoV-2 3CL^pro^ resulted in the interaction of 11 peptides with key-residues in the substrate-binding pocket of the enzyme. Among the 11 peptides, KFVPKQPNMIL showed interactions with key 3CL^pro^ catalytic residues, most importantly, the CYS-HIS dyad (Figure 3). Conventional hydrogen bonds were observed with CYS145, GLU166, GLN189, a Pi-Sigma bond with HIS41, and Pi-sulfur, and alkyl bonds were observed with MET49 (Table 2). The predicted ZDOCK score and E_RDOCK of the KFVPKQPNMIL-3CL^pro^ docked complex were 11.54 and −16.149 kcal/mol, respectively.

### Comparative Study of Peptide Interactions With RBD and 3CL^pro^ of β-Coronaviruses

**Figure 4:**
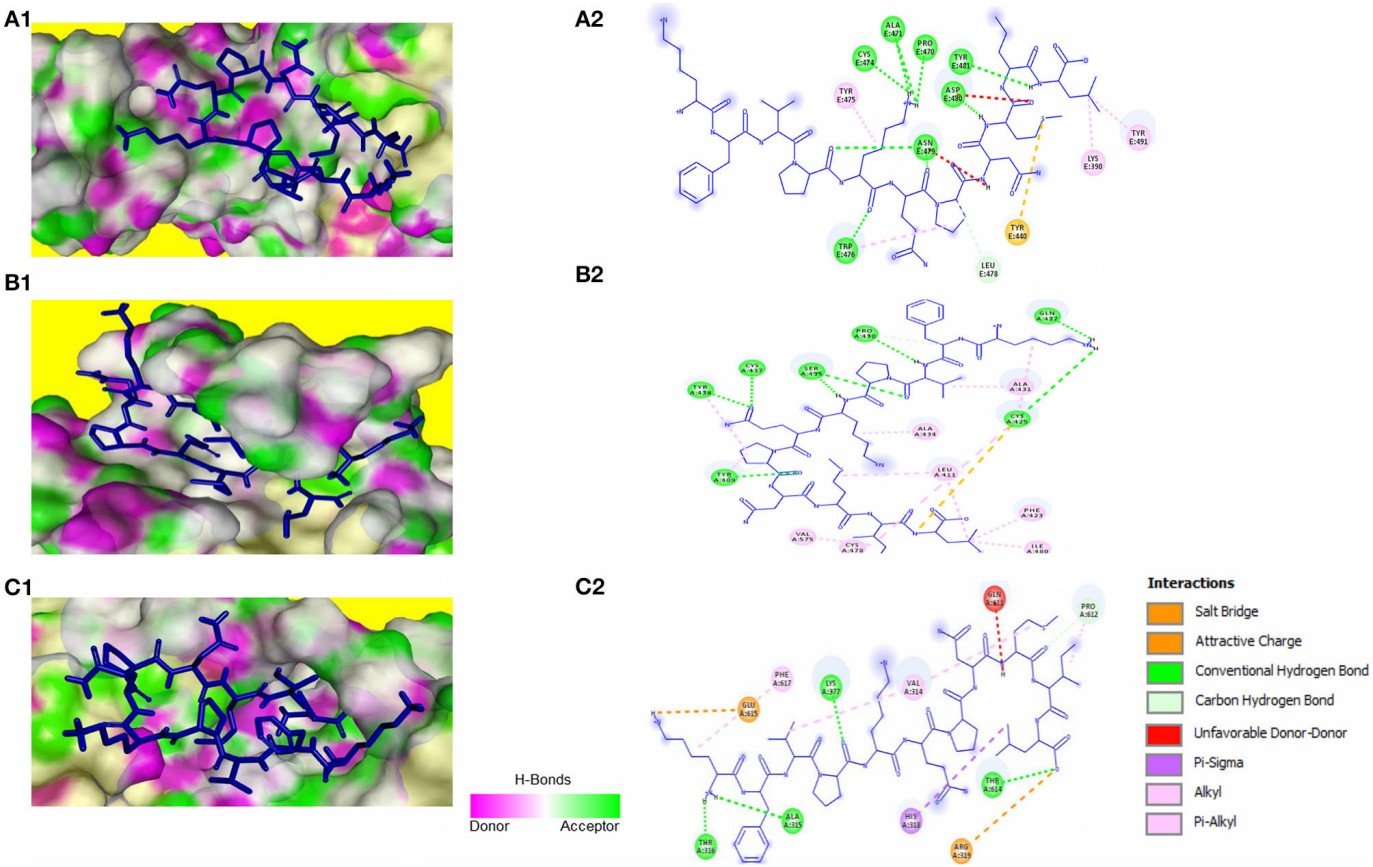
KFVPKQPNMIL interaction with RBD of SARS-CoV **(A)**, MERS-CoV **(B)**, and HCoV-HKU1 **(C)**. 3D illustration of complex between KFVPKQPNMIL-SARS-CoV RBD **(A1)**, KFVPKQPNMIL-MERS-CoV RBD **(B1)**, and KFVPKQPNMIL-HCoV-HKU1 RBD **(C1)**. RBD surface is represented by H-bond donor and acceptor atoms. 2D representation of different bonds formed during KFVPKQPNMIL interaction with RBD of SARS-CoV **(A2)**, MERS-CoV **(B2)**, and HCoV-HKU1 **(C2)**.

The peptide KFVPKQPNMIL showed conventional hydrogen bonds with key residues in the RBM of SARS-CoV S1 glycoprotein including ASN479 and ASP480. Comparatively, a higher affinity of the peptide toward SARS-CoV RBD was observed than that of SARS-CoV-2 with a predicted ZDOCK score of 12.14 and E_RDOCK of −11.64 kcal/mol (Table 3). Similarly, the conventional hydrogen bond between LEU11 and THR614, carbon hydrogen bond between MET9 and PRO612, and a salt bridge between LYS1 and GLU615 were observed in the KFVPKQPNMIL-HCoV-HKU1 RBD complex. Conventional hydrogen bond interactions were observed between the peptide and MERS-CoV RBD residues, CYS425, GLN427, PRO430, SER435, CYS437, and TYR438 situated outside the RBM (Figure 4). The predicted binding energies of the KFVPKQPNMIL-HCoV-HKU1 RBD complex and KFVPKQPNMIL-MERS-CoV RBD complex were −16.47 and −26.8 kcal/mol, respectively.

**Table 3:** Non-bond interactions between KFVPKQPNMIL and target protein residues of β-coronaviruses.

**Viruses**	**Peptidyl residue**	**Target protein residue**	**Interaction type**	**ZDOCK Score**	E_RDOCK^*^ (kcal/mol)	**Peptidyl residue**	**Target protein residue**	**Interaction type**	**ZDOCK Score**	E_RDOCK^*^ (kcal/mol)
	**Receptor Binding Domain (RBD)**					**3-Chymotrypsin like protease (3CL**^****pro****^**)**				
SARS-CoV	GLN6	ASN479	Conventional hydrogen			MET9	HIS41	Pi-Alkyl		
	MET9	ASP480	Conventional hydrogen	12.14	−11.64	PRO7	CYS145	Conventional hydrogen	9.94	−17.45
	LYS5	PRO470	Conventional hydrogen			VAL3	MET49	Alkyl		
	LYS5	CYS474	Conventional hydrogen			LEU11	MET165	Alkyl		
	LYS5	TRP476	Conventional hydrogen			MET9	GLU166	Conventional hydrogen		
	MET9	TYR440	Pi-Sulfur			LYS1	GLN189	Conventional hydrogen		
MERS-CoV	PRO7	TYR409	Conventional hydrogen			GLN6	HIS41	Conventional hydrogen		
	LYS1	CYS425	Conventional hydrogen	11.1	−26.80	PRO7	CYS145	Conventional hydrogen	12.8	−22.85
	VAL3	PRO430	Conventional hydrogen			GLN6	CYS148	Conventional hydrogen		
	VAL3	SER435	Conventional hydrogen			LYS5	LEU49	Conventional hydrogen		
	GLN6	CYS437	Conventional hydrogen			LYS5	TYR54	Conventional hydrogen		
	ILE10	CYS478	Alkyl			PRO7	HIS166	Pi-Alkyl		
	LEU11	ILE480	Alkyl			PRO4	GLU169	Conventional hydrogen		
	ILE10	VAL575	Alkyl			LYS1	HIS194	Conventional hydrogen		
H-CoV-HKU1	MET9	PRO612	Carbon hydrogen			LYS5	HIS41	Pi-Alkyl		
	LEU11	THR614	Conventional hydrogen	9.76	−16.47	GLN6	CYS145	Conventional hydrogen	11.12	−15.22
	LYS1	GLU615	Salt bridge			PRO7	CYS142	Alkyl		
	LYS1	PHE617	Pi-Alkyl			LYS5	CYS44	Conventional hydrogen		
						LYS5	TYR54	Conventional hydrogen		
						PRO4	GLU166	Conventional hydrogen		
						VAL3	LEU167	Alkyl		
						VAL3	VAL190	Conventional hydrogen		

* *E_RDOCK, Binding energy*.

**Figure 5:**
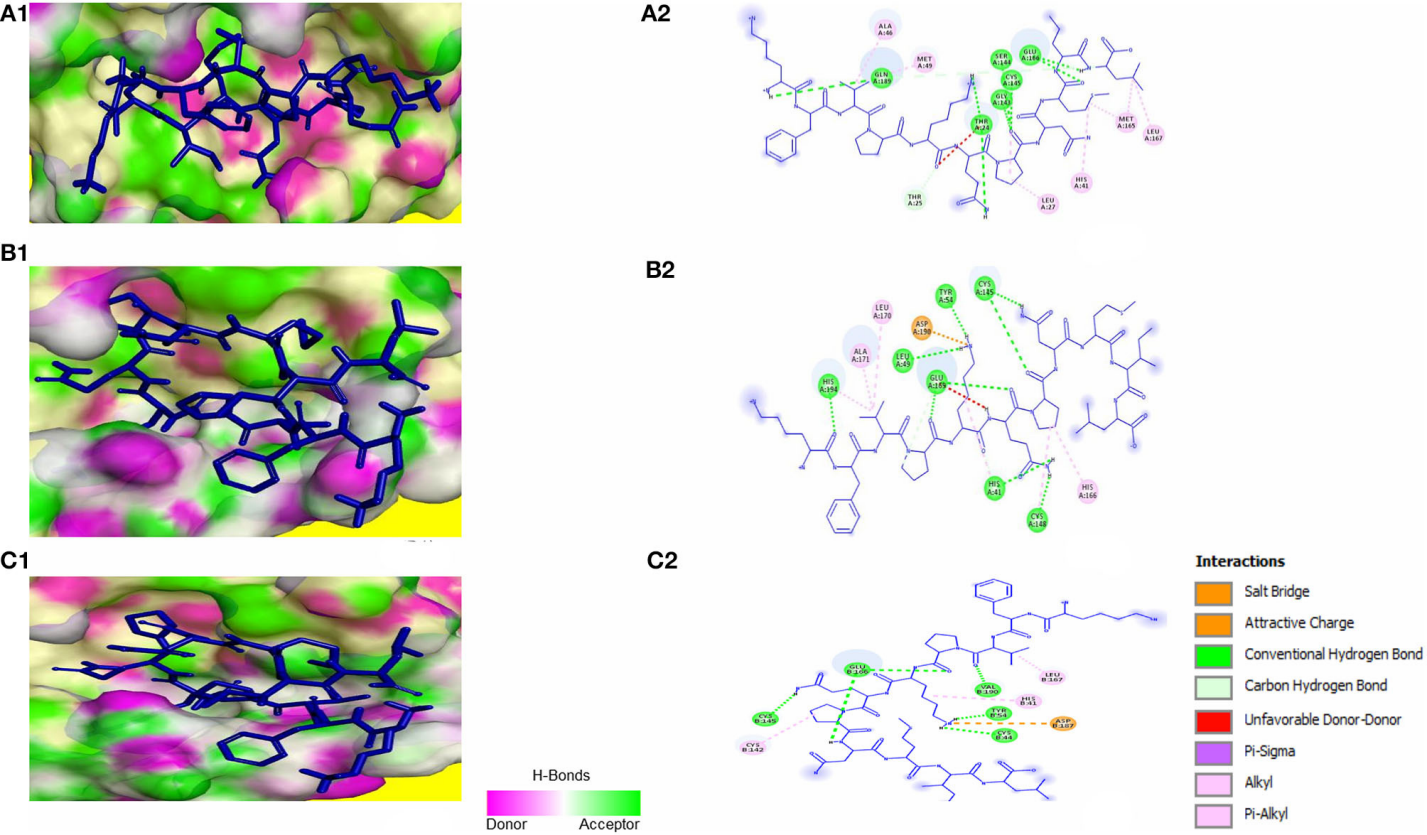
KFVPKQPNMIL interaction with 3CL^pro^ of SARS-CoV **(A)**, MERS-CoV **(B)**, and HCoV-HKU1 **(C)**. 3D illustration of complex between KFVPKQPNMIL-SARS-CoV 3CL^pro^ **(A1)**, KFVPKQPNMIL-MERS-CoV 3CL^pro^ **(B1)**, and KFVPKQPNMIL-HCoV-HKU1 3CL^pro^ **(C1)**. 3CL^pro^ surface is represented by H-bond donor and acceptor atoms. 2D representation of different bonds formed during KFVPKQPNMIL interaction with 3CL^pro^ of SARS-CoV **(A2)**, MERS-CoV **(B2)**, and HCoV-HKU1 **(C2)**.

Molecular docking of KFVPKQPNMIL with 3CL^pro^ of SARS-CoV, MERS-CoV, and HCoV-HKU1 revealed strong interactions between the peptide and CYS-HIS catalytic dyad of all three viral main proteases (Figure 5). Additionally, strong covalent and electrostatic interactions were observed between peptidyl residues and active site gating residues of the main protease. The predicted E_RDOCK of KFVPKQPNMIL interaction with 3CL^pro^ of SARS-CoV, MERS-CoV, and HCoV-HKU1 were −17.45, −22.85, and −15.22 kcal/mol respectively (Table 3).

## Discussion

Protein source of the predicted antiviral peptides includes β-conglycinin, proglycinin, seed maturation protein, lectin, and uncharacterized soy protein. Previous studies on biological activities of peptides released during soy fermentation have identified glycinin and β-conglycinin derived peptides with strong antimicrobial properties (Singh et al., 2019). The fact that β-conglycinin and glycinin are significant sources of bioactive peptides during soy fermentation has attracted most researchers to study the antimicrobial activity of these peptides (Tsuruki et al., 2003; Matemu et al., 2011). However, peptides from other proteins found in soybeans can exhibit antiviral activity. Some soy proteins specifically bind to glycosylated molecules and exert antiviral activity by interacting with viral envelope glycoproteins (Mitchell et al., 2017; Mazalovska and Kouokam, 2018). Peptides derived from these soy proteins can have an increased affinity for viral envelope proteins or can be potential inhibitors of viral replication (Keyaerts et al., 2007). The calculated pI of KFVPKQPNMIL was 10.00 indicating a positive charge of the peptide. It has been proposed that a positively charged peptide/molecule (pI > pH) presents a greater potential for interaction and inhibition of critical SARS-CoV-2 proteins (Gordon et al., 2020). With a GRAVY value of −0.07, KFVPKQPNMIL was predicted as slightly hydrophobic in nature. Hydrophilic nature is necessary for peptide solubility and interaction with viral glycoproteins (Edwards et al., 2016). In addition, hydrophilic peptides have been observed to be non-toxic in comparison to highly hydrophobic peptides (Yin et al., 2012).

Computational docking is widely used in drug discovery to quickly screen and identify compounds of therapeutic importance having strong affinities toward protein or enzyme-linked drug targets (Pinzi and Rastelli, 2019). The target proteins in this study are the RBD of SARS-CoV-2 spike S1 glycoprotein, responsible for attachment of the virus to the human receptor ACE2 protein, and the viral proteolytic enzyme 3CL^pro^ that is important for viral replication. Within the spike protein RBD, a structure motif called receptor binding motif (RBM) (SER438-GLN506) has been identified to be directly involved in SARS-CoV-2 interaction and subsequent attachment with ACE2 receptor (Lan et al., 2020). Furthermore, five RBM residues viz., LEU455, PHE486, GLN493, SER494, and ASN501 are reported to be conserved and play a critical role in the eventual binding of the virus to ACE2 for cell entry. Among them, GLN493 is the most critical residue for ACE2 recognition. The interaction of inhibitory peptidyl residues with these amino acids is expected to impede the attachment of SARS-CoV-2 spike protein to ACE2 receptor (Wan et al., 2020). Conventional hydrogen bond interactions between KFVPKQPNMIL and two RBM residues, GLN493, SER494, and an alkyl interaction between the peptide and LEU455 of the RBM indicates the potential role of KFVPKQPNMIL in binding with SARS-CoV-2 S1 glycoprotein. Considerable binding interactions between KFVPKQPNMIL and S1 RBD residues may lead to the competitive inhibition of ACE2-S1 RBD interactions. This may result in impeded attachment of the virus to the host ACE2 receptor. Hydrogen bonds are believed to facilitate affinity of the ligand toward the receptor, and their strength is directly related to the enhanced affinity (Chen et al., 2016). Additionally, non-covalent interactions such as alkyl, Pi-sigma, and Pi-sulfur help in ligand binding with receptor catalytic site through the transfer of charge between atoms (Arthur and Uzairu, 2019).

A ~800 kDa polypeptide is translated from the β-coronavirus genome during viral replication and is proteolytically cleaved by two major viral enzymes, papain-like protease (PL^pro^) and 3CL^pro^ encoded by the open reading frame 1(Zhu et al., 2020). Proteolysis of the polypeptide by 3CL^pro^ results in the generation of various non-structural proteins (NSPs) that are essential for viral replication (Anand et al., 2003). The 3CL^pro^ of all known coronaviruses, including the novel SARS-CoV-2 share sequence homology and substrate conservation making it an ideal target for broad spectrum inhibitor strategies (Berry et al., 2015). Additionally, the critical role of 3CL^pro^ in viral replication and the presence of 3CL^pro^ gene in the excessively variable 3' end of SARS-CoV-2 genome have made this enzyme a critical target for SARS-CoV-2 inhibitor screening (Needle et al., 2015; Tahir ul Qamar et al., 2020). The active site of the 3CL^pro^ enzyme is located in a chymotrypsin-like fold containing the CYS-HIS catalytic dyad, where the HIS41 acts as a proton acceptor, and the CYS144/145 undergoes a nucleophilic attack on the carbonyl carbon of the substrate (Yang et al., 2005, 2006). The viral polypeptide is cleaved at glutamine residue via the CYS-HIS dyad, where the cysteine thiol functions as the nucleophile during catalysis (Anand et al., 2003). Residues apart from the CYS-HIS dyad provide an opening gate to the active site for substrate binding (Yang et al., 2003). Although the catalytic active 3CL^pro^ is a dimer, yet solvent-exposed CYS-HIS dyads in the dimer are symmetrically located at opposite edges of the substrate-binding cleft. This suggests that the CYS-HIS dyads of individual monomers work independently in the protein dimer (Shi et al., 2008; Macchiagodena et al., 2020). Among the 11 peptides that demonstrated interactions with catalytic residues of 3CL^pro^, KFVPKQPNMIL showed considerable affinities toward the catalytic CYS-HIS dyad along with other active site residues GLU166 and GLN189. The presence of such binding interactions between KFVPKQPNMIL and 3CL^pro^ may lead to blocking of the enzyme's active catalytic pocket, impeding the functionality of 3CL^pro^, consequently resulting in regulation of viral RNA replication and inhibition of viral proliferation.

Interestingly, binding affinities were observed for KFVPKQPNMIL with critical residues of both RBD and 3CL^pro^.The peptide, KFVPKQPNMIL was identified from soy lectin protein. Lectins are reported to have affinity for glycosylated molecules and are known to bind to viral envelope glycoproteins, causing the inhibition of viral attachment with host receptor (Mitchell et al., 2017; Mazalovska and Kouokam, 2018). The soybean derived N-acetylgalactosamine (GalNAc) binding lectins have shown potential in suppressing infection of macrophages by human immunodeficiency virus (Zhou et al., 2017). Apart from inhibition of viral attachment, plant lectins such as mannose-binding lectins have demonstrated inhibition of SARS-CoV replication by interfering with specific targets in the viral replication cycle (Keyaerts et al., 2007). Thus, there is a promising potential of KFVPKQPNMIL to express dual roles in inhibiting SARS-CoV-2 attachment to human ACE2 receptors and in inhibiting its replication cycle.

Phylogenetically, coronaviruses are divided into four genera, namely α-, β-, γ-, and δ-coronaviruses. Among these, β-coronaviruses, including SARS-CoV in group B and MERS-CoV in group C are zoonoses that are associated with a severe respiratory infection, leading to pneumonia, acute respiratory distress syndrome (ARDS), and ultimately death (Qian et al., 2015). HCoV-HKU1, belonging to group A of β-coronaviruses is found in humans worldwide and can occasionally cause severe respiratory diseases, such as pneumonia in elderly, very young, and immune-compromised individuals (Gralinski and Baric, 2015). The novel SARS-COV-2 virus shares high genetic homology with SARS-CoV and is associated with severe respiratory infection strikingly similar to that caused by SARS-CoV, MERS-CoV, and HCoV-HKU1 (Du et al., 2009; Zaki et al., 2012; Qian et al., 2015). The RBD of all human infecting β-coronaviruses except HCoV-OC43 lie in the C Domain of the S1 glycoprotein (Lin et al., 2014; Qian et al., 2015). A variety of RBM exist in the C domain that recognize specific determinants of host cells such as ACE2, or dipeptidyl peptidase 4 (DPP4), that act as receptors for different coronaviruses (Qian et al., 2015). Structural comparison of SARS-CoV-2 3CL^pro^ (PDB ID: 6LU7) and 3CL^pro^ of SARS-CoV (PDB ID: 1UK4) revealed that there is a difference of only 12 amino acids between the two proteases, with carbon atoms of the varying residues located at a minimum of 1 nm distance from the catalytic site of the enzyme. Thus, residues in the substrate-binding site of the two enzymes have very high similarity (RMSD = 0.99 Å) (Macchiagodena et al., 2020). Knowledge of conserved RBM location in S1 glycoprotein and high 3CL^pro^ sequence homology and substrate conservation between β-coronaviruses can be explored to develop broad-spectrum inhibitors against viral infections to prevent the damage caused by the emergence of future SARS-like respiratory illnesses (Padhi et al., 2020). KFVPKQPNMIL showed interactions with RBD of SARS-CoV, including conventional hydrogen bond interactions with critical residues responsible for receptor attachment. Although high binding energy (−26.8 kcal/mol) between KFVPKQPNMIL and MERS-CoV RBD was observed, the peptide did not interact with critical RBM residues responsible for receptor attachment. This may be due to the difference in target receptor specificity of MERS-CoV RBM (DPP4) than SARS-CoV and SARS-CoV-2 (ACE2) (Li, 2015). However, the peptide demonstrated strong interactions with 3CL^pro^ catalytic CYS-HIS dyad and gating residues of all four β-coronaviruses with the highest binding energy of −22.85 kcal/mol observed for KFVPKQPNMIL-MERS-CoV 3CL^pro^ complex.

Due to the rapid spread of SARS-CoV-2 infection, lack of a potent cure as of present and large-scale public health care measures enforced to reduce viral transmission rates, the focus of the general public has been on healthier foods and nutraceuticals with immune-boosting properties (Aday and Aday, 2020). Increase in consumption of functional foods and nutraceuticals has been observed in individuals with high infection risk, including health care professionals (Furlong, 2020). Such natural ingredients based preventive healthcare have been considered to be effective against SARS and influenza virus in the past (Ayseli et al., 2020). Increased interest in functional foods and nutraceuticals based natural therapies has resulted in a change in the health paradigm from a curative to a preventive model (Ayseli et al., 2020). The present study proposes antiviral peptide enriched soy cheese as natural therapeutic offering prophylaxis against viral infections. The soy cheese can be consumed as a functional food for the prevention of viral infections. Alternatively, antiviral peptides can be produced as nutraceutical protein hydrolysates from soy milk fermentation by exploiting the activity of *Lb. delbrueckii* WS4 proteinases. Large scale production of peptides as nutraceuticals can be achieved by various methods including: fractionization and isolation of target peptide from protein hydrolysate produced during fermentation by microorganisms (Agyei and Danquah, 2011), chemical synthesis of peptides using chemical reagents to mediate peptide bond formation (Perez Espitia et al., 2012; Brimble et al., 2015), and production of therapeutic peptides via recombinant DNA technology (Kyle et al., 2012; Long et al., 2012). Further studies on food-derived peptides such as KFVPKQPNMIL that are capable of interacting with multifarious multi-viral drug targets can lead to development of prophylactics against viral illnesses such as SARS-CoV-2.

## Conclusions

Countries all around the world have severely been affected by the COVID19 pandemic caused by the novel SARS-CoV-2 virus. The lack of therapeutic strategies to combat this viral infection has initiated a global race to quickly find a prophylactic measure to prevent its further spreading by acting on its target proteins. Fermented soy-derived peptides have previously demonstrated activity against various viruses, including SARS-CoV that was responsible for the 2003 SARS outbreak. Herein, molecular docking studies of predicted antiviral peptides released during the production of soy cheese using *Lb. delbrueckii* WS4 were performed. The lectin derived peptide KFVPKQPNMIL successfully interacted with critical RBD residues of SARS-CoV-2 spike protein and important catalytic residues of the viral proteolytic enzyme 3CL^pro^. Similar interactions of KFVPKQPNMIL with critical residues of the target proteins were observed for other β-coronaviruses. KFVPKQPNMIL is the first food-derived peptide that has shown interaction with two functionally different and clinically significant SARS-CoV-2 proteins. The findings of this study could be used for *in vitro* and *in vivo* investigations to neutralize the infection of the deadly SARS-CoV-2 and similar viruses. Moreover, soy cheese fermented using *Lb. delbrueckii* WS4 and its aqueous extract containing various antiviral peptides can be used as a prophylactic to prevent SARS-CoV-2 and other viral infections.

## Data Availability Statement

The raw data supporting the conclusions of this article will be made available by the authors, without undue reservation.

## Author Contributions

AR and SS contributed to conception, design of the study, and finalization of manuscript. RC developed the cheese product and did a major part of bioinformatics analysis and wrote the first draft of the manuscript. RC and LP did the proteomics analysis. SP, LP, and MA assisted in bioinformatics analysis, development of cheese products, and wrote sections of the manuscript. All authors contributed to manuscript revision, read, and approved the submitted version.

## Conflict of Interest

The authors declare that the research was conducted in the absence of any commercial or financial relationships that could be construed as a potential conflict of interest.
